# Clinical Outcomes of Peptide Receptor Radionuclide Therapy in Japanese Patients with Metastatic Rectal Neuroendocrine Tumors

**DOI:** 10.3390/cancers18081268

**Published:** 2026-04-16

**Authors:** Takeshi Iizuka, Noritoshi Kobayashi, Damian Wild, Felix Kaul, Hiroaki Suzuki, Kengo Maehara, Naoki Okubo, Sho Tsuyuki, Shoko Takano, Yusuke Kurita, Masato Yoneda, Yasushi Ichikawa

**Affiliations:** 1 Department of Oncology, Yokohama City University Hospital, Yokohama 236-0004, Japan; iizuka.tak.ax@yokohama-cu.ac.jp (T.I.);; 2 Division of Nuclear Medicine, University Hospital of Basel, 4031 Basel, Switzerland; 3 Department of Nuclear Medicine, Yokohama City University Hospital, Yokohama 236-0004, Japan; 4 Department of Gastroenterology and Hepatology, Yokohama City University Hospital, Yokohama 236-0004, Japan

**Keywords:** rectal neuroendocrine tumor, PRRT, [^177^Lu]Lu-DOTA-TATE, SSTR, NSE, progression-free survival

## Abstract

**Background/Purpose**: Rectal neuroendocrine tumors (NETs) with distant metastases are uncommon, and evidence supporting the effectiveness of peptide receptor radionuclide therapy (PRRT) in this population remains limited, particularly in Asian cohorts. This study aimed to evaluate the clinical role and real-world outcomes of PRRT in Japanese patients with somatostatin receptor-positive metastatic rectal NETs. **Methods**: We retrospectively analyzed 20 patients with metastatic rectal NETs who underwent PRRT at the University Hospital Basel (Switzerland) and Yokohama City University Hospital (Japan) between April 2015 and May 2023. The primary endpoint was progression-free survival (PFS), and secondary endpoints included disease control rate (DCR), overall response rate (ORR), overall survival (OS), and adverse events (AEs). Exploratory subgroup analyses were performed according to clinical characteristics, including changes in serum neuron-specific enolase (NSE). **Results**: The median PFS was 18.9 months (95% CI, 13.5–24.3), and the median OS was 30.3 months (95% CI, 18.9–41.7). The DCR was 80.0%, and the ORR was 15.0%. Treatment responses included partial response in 3 patients, stable disease in 13, progressive disease in 3, and not evaluable in 1. The most common AEs were lymphopenia and anemia, and no secondary malignancies were observed. No clinical factors were significantly associated with PFS; however, higher baseline NSE levels showed a trend toward shorter PFS. Patients with post-treatment declines in NSE showed more favorable treatment responses. **Conclusions**: PRRT may provide durable disease control with a favorable safety profile in Japanese patients with metastatic rectal NETs. However, these findings should be interpreted with caution given the small sample size and heterogeneous patient population. Baseline NSE elevation and early post-treatment declines may serve as potential prognostic indicators. These results are hypothesis-generating and warrant validation in larger, multicenter studies.

## Simple Summary

Peptide receptor radionuclide therapy (PRRT) is an established treatment for advanced neuroendocrine tumors; however, evidence in metastatic rectal neuroendocrine tumors remains limited, particularly in Asian populations. In this study, we evaluated 20 Japanese patients with somatostatin receptor (SSTR)-positive metastatic rectal neuroendocrine tumors treated with PRRT at two specialized centers in Japan and Switzerland. PRRT demonstrated favorable clinical outcomes, with a median progression-free survival of 18.9 months and overall survival of 30.3 months. Treatment was generally well tolerated, with a low incidence of severe adverse events. Patients with elevated baseline neuron-specific enolase (NSE) showed a trend toward poorer outcomes, suggesting its potential role as a prognostic biomarker. These findings provide real-world evidence supporting the safety and efficacy of PRRT in this rare patient population and may help inform clinical decision-making.

## 1. Introduction

Rectal neuroendocrine tumors (NETs) are reported to constitute a minority (approximately 1–2%) of all rectal tumors [1]. With the increase in colonoscopies for colorectal cancer screening, the number of reported cases of rectal NETs is increasing [2]. Rectal NETs account for 14–29.6% of all gastroenteropancreatic (GEP) NETs [3,4,5], but rectal NETs with lymph node or multi-organ metastases are rare, occurring in approximately 5–15% of cases [1,6,7].

The annual incidence rate of GEP-NET in Japan was 0.70 per 100,000 for pancreatic NET and 2.84 per 100,000 for gastrointestinal neuroendocrine neoplasms [8]. Tumors in the duodenum, appendix, and rectum were primarily locally confined, whereas tumors in the esophagus, stomach, and colon tended to show distant metastasis. For rectal NET, 32.4% of patients had metastasis and both lymph nodes and the liver were frequent sites of metastasis [8].

The median overall survival (OS) for distant metastases across the entire GEP-NET cohort has been reported as 34 months (approximately 2.8 years). For distant metastases originating in the rectum, median OS was approximately 11 months (range 8.8–13.2 months) [9]. The prognosis for metastatic rectal NETs may be worse compared to NETs originating in multiple organs [6].

Peptide receptor radionuclide therapy (PRRT), a form of systemic radiotherapy, allows the targeted delivery of radionuclides to tumor cells expressing high levels of somatostatin receptors (SSTRs) [10]. PRRT is generally effective in tumors with sufficient somatostatin receptor expression, whereas its efficacy may be limited in tumors with low SSTR expression due to inadequate radiotracer uptake. It has shown significant promise for treatment of advanced, low- to intermediate-grade NETs [11,12,13]. Developed using radionuclides such as Yttrium-90 (^90^Y) and Lutetium-177 (^177^Lu), its efficacy in treating NETs has been extensively demonstrated [14]. Currently, PRRT is a standard therapy for the treatment of unresectable NETs. During the early study period, PRRT was not yet approved for reimbursement in Japan; therefore, selected patients were referred to the University Hospital Basel (Switzerland), a specialized center for PRRT, enabling access to this treatment through international collaboration.

The European Neuroendocrine Tumor Society (ENETS) has updated its guidelines for the management of rectal NETs in 2023 [15], but there are limited treatment options for rectal NETs with distant metastases. ENETS recommends PRRT for “well-differentiated (G1–2) GEP-NET” that is SSTR-positive and has progressed or metastasized. Although it is not a standard treatment, it has been noted as a possible option following chemotherapy or therapy with somatostatin analogues (SSAs) [15]. Furthermore, the National Comprehensive Cancer Network (NCCN) Guidelines explicitly state that PRRT with [^177^Lu]Lu-DOTA-TATE may be considered in patients with SSTR-positive Stage IV disease. SSA (lanreotide/octreotide) is the first-line choice for disease control in SSTR-positive, low-volume advanced/metastatic cases, while PRRT is positioned as a recommended option following SSA.

Previous studies have suggested that the tumor response to PRRT may be lower in rectal NETs compared with NETs arising from other primary sites [16]. In addition, the incidence of rectal NETs varies geographically, with a higher prevalence reported in Asian populations than in Western countries [17]. However, clinical evidence regarding the effectiveness of PRRT, specifically for SSTR-positive metastatic rectal NETs, remains limited, and most available data are derived from heterogeneous GEP-NET cohorts rather than disease-specific analyses.

Therefore, the present study aimed to retrospectively evaluate the real-world clinical outcomes and treatment positioning of PRRT in Japanese patients with SSTR-positive metastatic rectal NETs treated at two specialized centers.

## 2. Materials and Methods

### 2.1. Patients

This was a retrospective, two-center study conducted at the University Hospital Basel (Switzerland) and Yokohama City University Hospital (Japan). We evaluated 20 patients who received at least one session of PRRT for the treatment of SSTR-positive metastatic rectal NETs at the University Hospital Basel and Yokohama City University Hospital between April 2015 and May 2023. All patients included in this study were Japanese, although PRRT was performed either in Japan or in Switzerland depending on treatment availability during the study period. Patients who underwent PRRT in Switzerland were referred from Japan during the early study period, when PRRT was not yet approved for reimbursement domestically. After completion of treatment, patients returned to Japan for follow-up and subsequent clinical management. Patients were followed up to death or study closure date of December 2024.

Indications for PRRT include a pathologically confirmed neuroendocrine tumor of Grade 1 or 2 (Grade 3 is indicated only for well-differentiated NET G3 cases). Long-acting SSAs were discontinued prior to treatment when clinically indicated. Somatostatin receptor scintigraphy (SRS) with [^111^In]In-pentetreotide or [^68^Ga]Ga-DOTA-TOC PET/CT was performed. [^68^Ga]Ga-DOTA-TOC PET/CT scans are not yet approved in Japan and were used only in a limited number of cases.

The inclusion criteria were Eastern Cooperative Oncology Group (ECOG) performance status < 2. Furthermore, patients were required to travel to Switzerland for treatment. Adequate bone marrow, renal and hepatic functions were required (white blood cell count > 3000/μL, hemoglobin count > 8.0 g/dL, platelet count > 90,000/L, creatinine < 1.5, bilirubin level < 3 × upper limit).

Since this was a retrospective observational study, only preexisting medical records were reviewed. The present study was approved by the Institutional Review Board of our institution (approval number F230900033). All authors have approved the final manuscript.

### 2.2. Treatment Regimen

This study included 20 patients who underwent PRRT for SSTR-positive metastatic rectal NETs between April 2015 and May 2023 at the University Hospital Basel and Yokohama City University Hospital. PRRT was administered using [^177^Lu]Lu-DOTA-TATE, [^177^Lu]Lu-DOTA-TOC, or [^90^Y]Y-DOTA-TOC. Induction PRRT was generally delivered over three to four cycles at 8–16-week intervals.

Each cycle involved administration of [^177^Lu]Lu-DOTA-TATE at activities ranging from 3.7 to 7.4 GBq. At the University Hospital Basel, [^177^Lu]Lu-DOTA-TOC was used in all cases, with injected activities ranging from 3.7 to 5.5 GBq per cycle. In three patients, [^90^Y]Y-DOTA-TOC was administered concomitantly. Treatment at the University Hospital Basel generally consisted of three cycles, with [^177^Lu]Lu-DOTA-TOC administered during the first and third cycles and [^90^Y]Y-DOTA-TOC administered only during the second cycle. Injected activities of [^90^Y]Y-DOTA-TOC ranged from 3.0 to 4.8 GBq per cycle.

At both institutions, treatment was discontinued if predefined discontinuation criteria were met, and injected activity reductions were implemented when clinically indicated.

### 2.3. PRRT Treatment Protocol

Each PRRT cycle was administered with standard premedication (granisetron 2 mg and dexamethasone 8 mg), followed by a renal protective amino-acid infusion containing 25 g lysine and 25 g arginine in 1 L of normal saline. The amino-acid solution was started 30 min before the administration of [^177^Lu]Lu-DOTA-TOC or [^90^Y]Y-DOTA-TOC and continued for approximately 3 h at the University Hospital Basel.

In Japan, PRRT was performed according to the approved protocol for [^177^Lu]Lu-DOTA-TATE (Lutathera^®^, Novartis, Basel, Switzerland), consisting of four 7.4 GBq infusions every 8 weeks. Premedication with palonosetron 0.75 mg was routinely used, and the same amino-acid infusion protocol for renal protection was applied. Patients were admitted to a dedicated radiopharmaceutical therapy ward in accordance with Japanese radiation-safety regulations.

### 2.4. Follow-Up

Patients were evaluated before PRRT and after the 2nd and 4th treatment cycles, as appropriate, typically within six months after the final cycle. Assessments included evaluation of clinical symptoms, laboratory tests, and cross-sectional imaging with CT or MRI. Laboratory analyses assessed the degree of myelosuppression, renal and hepatic function, and serum tumor markers (NSE and Pro-GRP). Patients were followed until death or the study cutoff date of December 2024.

### 2.5. Endpoints

The primary endpoint was progression-free survival (PFS), defined as the interval from the initiation of PRRT to radiological disease progression or death from any cause, whichever occurred first. Secondary endpoints included the disease control rate (DCR), overall response rate (ORR), overall survival (OS), and treatment-related adverse events (AEs). Tumor response was evaluated according to the Response Evaluation Criteria in Solid Tumors (RECIST) version 1.1, based on contrast-enhanced CT or MRI at baseline and at predetermined follow-up intervals. Radiological assessments were generally performed every 3–6 months during follow-up. All imaging studies were reviewed by experienced radiologists and nuclear medicine physicians at each participating institution (University Hospital Basel and Yokohama City University Hospital), who were involved in routine clinical care.

We evaluated the association between PFS and the following variables. Continuous variables included age, Ki-67 index, baseline serum neuron-specific enolase (NSE) level, and the interval from initial diagnosis to initiation of PRRT. Categorical variables included sex (male vs. female), WHO differentiation grade (G1–G2 vs. G3), maximum diameter of liver metastases (≤30 mm vs. >30 mm), liver tumor burden (≤25% vs. >25%), therapy lines prior to PRRT (≤3 vs. >3), and treatment institution (Basel vs. Yokohama). The cutoff value for NSE (16.3 ng/mL) was defined based on the institutional upper limit of normal (0–16.3 ng/mL) and was used for subgroup analyses.

In addition, we examined the relationship between changes in tumor markers and treatment response in patients with decreased tumor markers.

### 2.6. Statistical Analysis

All statistical analyses were performed using IBM SPSS Statistics (version 29.0; IBM Corp., Armonk, NY, USA). Overall survival (OS) and progression-free survival (PFS) were estimated for the entire cohort using the Kaplan–Meier method, and differences between groups were assessed using the log-rank test.

Associations between clinical variables and PFS were evaluated using univariate Cox proportional hazards models. Continuous variables were analyzed as continuous variables, while selected clinical factors were dichotomized as indicated. Given the limited sample size, multivariate analysis was not performed.

Adverse events were graded according to the Common Terminology Criteria for Adverse Events (CTCAE) version 5.0.

## 3. Results

### 3.1. Study Population

A total of 20 patients with metastatic rectal NETs who underwent PRRT were included. Patient characteristics are summarized in Table 1. The median age at the start of PRRT was 63.5 years (range, 46–78), and 13 patients (65.0%) were male. The median Ki-67 index was 4.5% (range, 1–28.7%). Baseline tumor marker levels were as follows: NSE, 20.3 ng/mL (range, 9.7–21.7); Pro-GRP, 46.2 pg/mL (range, 26.3–79.9). According to WHO 2019 grading system, 3 patients (15.0%) had G1, 15 (75.0%) had G2, and 2 (10.0%) had G3 disease. All cases were non-functional. Krenning scores were 3 in 3 patients (15.0%) and 4 in 17 patients (85.0%). There were 13 primary lesion resections, of which 12 were R0 resections and 1 was R2 resection. Sites of distant metastases included the liver in 19 patients (95.0%), bone in 11 (55.0%), and lung in 4 (20.0%), lymph node metastases in 8 (40.0%).

### 3.2. Details of PRRT Treatment

This study included 20 patients who underwent PRRT for SSTR-positive metastatic rectal NETs between April 2015 and May 2023. Details of PRRT treatment are summarized in Table 2. The median number of treatment lines prior to PRRT was 5 (1–7). Eight patients who underwent PRRT before June 2021 received treatment at the University Hospital Basel. At University Hospital Basel, [^177^Lu]Lu-DOTA-TOC was used, whereas [^177^Lu]Lu-DOTA-TATE was used domestically. Treatment consisted of [^177^Lu]Lu-DOTA-TATE monotherapy in 12 patients (60.0%), [^177^Lu]Lu-DOTA-TOC monotherapy in 5 patients (25.0%) and [^177^Lu]Lu-DOTA-TOC and [^90^Y]Y-DOTA-TOC combination therapy in 3 patients (15.0%). In total, 16 patients were treated with SSA, 12 with everolimus, 5 with streptozocin, 2 with capecitabine plus temozolomide chemotherapy, 2 with capecitabine, and 2 with 5-Fluorouracil before PRRT. Local treatment included trans arterial chemoembolization (TACE) in three cases, radio frequency ablation (RFA) in one case, and heavy particle therapy in one case.

### 3.3. Efficacy Outcomes

**Figure 1:**
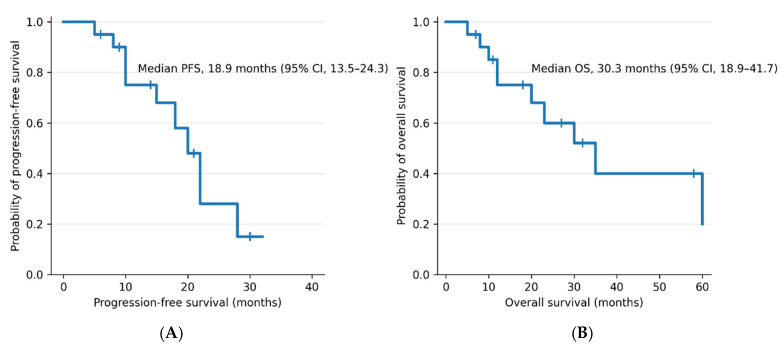
Kaplan–Meier curves for progression-free survival (PFS) and overall survival (OS) in patients with metastatic rectal NETs treated with PRRT. Tick marks indicate censored observations. (**A**) Progression-free survival. Median PFS was 18.9 months (95% CI: 13.5–24.3). (**B**) Overall survival. Median OS was 30.3 months (95% CI: 18.9–41.7).

The median PFS was 18.9 months (95% CI: 13.5–24.3), the median OS was 30.3 months (95% CI: 18.9–41.7), DCR was 80.0%, ORR was 15.0% (Table 3). Anti-tumor efficacy was partial response (PR) in 3 patients (15.0%), stable disease (SD) in 13 patients (65.0%), progressive disease (PD) in 3 patients (15.0%), not evaluable (NE) in 1 patient (5.0%) (Figure 1, Table 3). Tumor response was not evaluable in one patient due to insufficient radiological follow-up after PRRT.

All 20 patients were evaluated using univariate analyses to assess the association between PFS and the following factors: age, sex (male vs. female), WHO differentiation grade (G1–G2 vs. G3), Ki-67 index, baseline serum neuron-specific enolase (NSE) level, maximum diameter of liver metastases (≤30 mm vs. >30 mm), liver tumor burden (≤25% vs. >25%), interval from initial diagnosis to initiation of PRRT, number of therapy lines prior to PRRT (≤3 vs. >3), and treatment institution (Basel vs. Yokohama). In univariate analyses, no clinical or pathological variables were significantly associated with PFS; however, higher baseline NSE levels showed a trend toward shorter PFS (Table 4). Treatment institution was not associated with PFS.

### 3.4. The Relationship Between Tumor Markers and Anti-Tumor Effect

**Figure 2:**
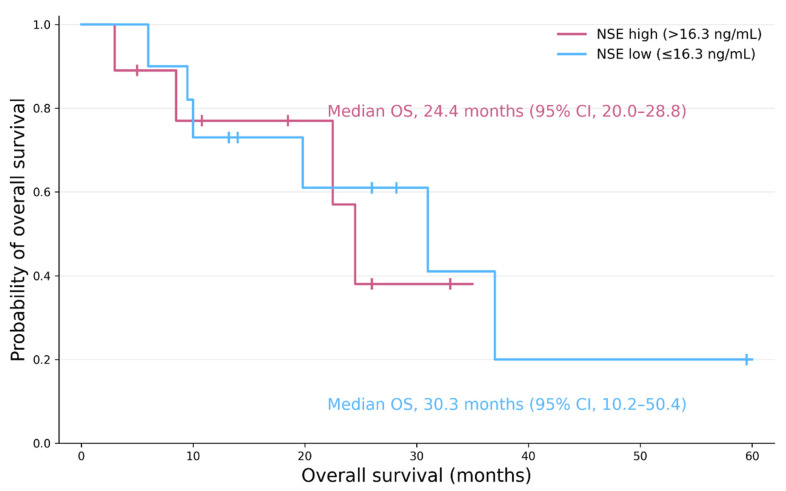
Overall survival according to baseline NSE level. Kaplan–Meier curves comparing patients with elevated baseline NSE (>16.3 ng/mL) and those with NSE within the normal range (≤16.3 ng/mL). Tick marks indicate censored observations. Median OS was 24.4 months (95% CI: 20.0–28.8) in the high-NSE group and 30.3 months (95% CI: 10.2–50.4) in the low-NSE group. There was no statistically significant difference between the two groups (log-rank test, *p* = 0.728).

**Figure 3:**
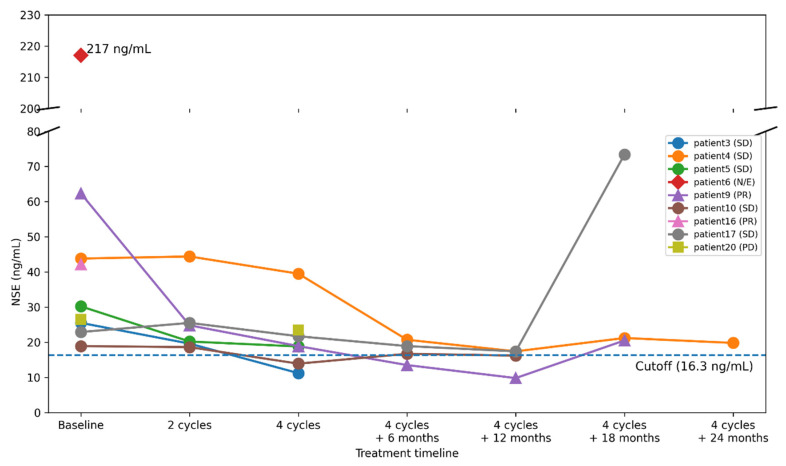
Changes in serum NSE levels during PRRT. Individual patient trajectories of serum NSE levels at baseline and following four cycles of PRRT are shown. Four cycles were selected as they represent the standard completion of induction PRRT and the primary timepoint for response assessment. Among patients with elevated baseline NSE levels (>16.3 ng/mL), seven exhibited a post-treatment decline, which was generally associated with partial response (PR) or stable disease (SD). The dashed line indicates the cutoff value for NSE (16.3 ng/mL). Marker shapes indicate treatment response: triangles (PR), circles (SD), squares (PD), and diamonds (N/E). A break in the y-axis is used to accommodate outlier values.

Nine patients had serum NSE levels above the upper normal limit (16.3 ng/mL), whereas none exceeded the Pro-GRP threshold (81.0 pg/mL) prior to initiation of PRRT. Among the nine patients with elevated NSE levels before PRRT, the median PFS was 20.7 months (95% CI: 9.7–31.6) and the median OS was 24.4 months (95% CI: 20.0–28.8). In contrast, among the 11 patients with NSE levels below the threshold prior to PRRT initiation, the median PFS was 17.0 months (95% confidence interval: 8.7–25.1), and the median OS was 30.3 months (95% CI: 10.2–50.4), noting the wide confidence intervals due to the limited number of events (Figure 2). Among patients with elevated NSE levels prior to PRRT, seven showed a decrease in NSE levels below the threshold after four cycles of PRRT (Figure 3). Treatment response in these seven cases was SD in five cases, PR in one case, and PD in one case.

### 3.5. Adverse Events of PRRT

Regarding AEs, hematologic toxicities such as lymphopenia and anemia were observed in 80% of all patients. Elevated AST/ALT, thrombocytopenia, and increased creatinine levels were also frequently observed. Serious adverse events (SAE), defined as Grade 3 or higher according to CTCAE version 5.0, included lymphopenia (15%), anemia (5%), and elevated bilirubin levels (5%).

Non-hematologic toxicities included nausea in 25% of patients and vomiting in 15% of patients (Table 5). Two patients discontinued PRRT during treatment: one patient developed G3 anemia, and in another patient, disease progression occurred after the first cycle of PRRT, with subsequent discontinuation due to hyperbilirubinemia caused by liver metastases.

Four patients required dose reductions during treatment. The single injected activity was reduced from 5.5–7.4 GBq of [^177^Lu]Lu-DOTA-TOC to 3.7 GBq. Reduction of injected activity was implemented in two patients due to renal dysfunction, in one patient due to extensive bone metastases (25% or more), and in one patient at the physician’s discretion. No cases of myelodysplastic syndrome or leukemia were observed during the follow-up period.

## 4. Discussion

In this retrospective study, we evaluated the clinical outcomes of PRRT in Japanese patients with metastatic rectal NETs. The median PFS was 18.9 months (95% CI: 13.5–24.3) and the median OS was 30.3 months (95% CI: 18.9–41.7). Patients with metastatic rectal NETs are known to have a poor prognosis [18], and robust evidence to guide optimal treatment strategies remains limited. In this context, our findings suggest that PRRT may achieve disease control in patients with metastatic rectal NETs, even in heavily pretreated clinical settings; however, these findings should be interpreted with caution given the heterogeneity of the study population.

In contrast, large international clinical trials have demonstrated favorable responses and sustained efficacy of PRRT in patients with SSTR expressing GEP-NETs. Reported median PFS values include 28.4 months in the NETTER-1 trial [12], 26.7 months in the Erasmus MC trial [13], and 22.8 months in the NETTER-2 trial [19]. A retrospective study of 27 patients with rectal NETs treated with PRRT reported partial responses in 70% of patients, stable disease in 26%, and median PFS of 29 months [20]. However, most of these studies included heterogeneous NET populations, and evidence focusing specifically on SSTR-positive metastatic rectal NETs remains limited.

In our cohort, the median PFS was 18.9 months, which was shorter than that reported in major PRRT trials. This difference may reflect real-world treatment sequencing, in which PRRT is often introduced after multiple prior systemic therapies. In addition, many patients in our cohort initiated PRRT at an advanced disease stage, frequently with high hepatic tumor burden and large liver metastases. These factors have been associated with poorer outcomes in previous studies and may have contributed to the relatively shorter PFS and OS observed in this study. Furthermore, PRRT was often administered as a later-line treatment in our cohort, reflecting its limited availability in Japan during the earlier study period. Taken together, the advanced disease status, high tumor burden, and delayed initiation of PRRT likely explain the modestly shorter survival outcomes compared with those reported in clinical trials.

Rectal NETs may represent a biologically distinct subgroup within GEP-NETs. Compared with pancreatic or midgut NETs, rectal NETs arise from hindgut-derived neuroendocrine cells and may exhibit different clinical behavior and treatment responses [12]. Previous studies have suggested that tumor shrinkage after PRRT may be less pronounced in rectal NETs than in NETs from other primary sites, and rectal origin has been reported as a factor associated with resistance to tumor shrinkage [14,16], including findings from our previous lesion-based analysis. Importantly, the present cohort represents an independent patient population distinct from that of the prior study. These findings are consistent with our results and support the need for disease-specific evaluation of PRRT in rectal NETs.

No significant associations were observed between PFS and age, sex, WHO differentiation grade, Ki-67 index, interval from diagnosis to initiation of PRRT, or overall liver tumor burden. In univariate analysis, higher baseline serum neuron-specific enolase (NSE) levels showed a trend toward shorter PFS, although this did not reach statistical significance. Elevated NSE may reflect underlying tumor aggressiveness rather than treatment responsiveness alone, whereas the size of the dominant liver lesion may have a greater prognostic impact than total hepatic tumor volume. Previous studies have suggested that treatment efficacy may be more strongly influenced by the maximum diameter of liver metastases than by overall hepatic tumor volume [13], although evidence specific to rectal NETs remains limited. Larger studies with longer follow-up are needed to clarify the prognostic relevance of these factors. Given the limited sample size and heterogeneity of the study cohort, these findings should be interpreted as exploratory. Although PRRT protocols varied between institutions, including the use of [^90^Y]Y-DOTA-TOC in a subset of patients, which differs from [^177^Lu]-based PRRT in terms of radiation characteristics and tissue penetration, all treatments were performed according to standard clinical practice, and treatment institution was not associated with PFS in univariate analysis.

Elevated NSE levels were observed in nine patients prior to initiation of PRRT. Although PFS did not differ from that of the overall cohort, the median OS in these patients (24.4 months; 95% CI, 20.0–28.8) showed a trend toward shorter survival, suggesting that elevated baseline NSE may reflect more aggressive tumor biology. Among these nine patients, seven exhibited a reduction in NSE levels after four cycles of PRRT, including cases with partial response and stable disease, indicating that early decreases in NSE may correlate with treatment response. In contrast, Pro-GRP levels remained within the normal range in all patients, consistent with the established biomarker profile of rectal NETs. While data on tumor markers and prognosis in metastatic rectal NETs are limited, our findings are consistent with prior reports suggesting that serum NSE may serve as a prognostic indicator in patients with GEP-NETs. Collectively, these results suggest that baseline NSE elevation may indicate poor prognosis, whereas early NSE reduction during PRRT may serve as a useful indicator of therapeutic response.

Serum NSE has been identified as an independent prognostic factor for overall survival in GEP-NETs [21], and levels exceeding 35 ng/mL have been associated with shorter PFS in patients undergoing PRRT [22]. Consistent with these reports, our findings suggest that elevated baseline NSE may indicate a poor prognosis, while an early decrease in NSE during treatment (e.g., within 2–4 cycles of PRRT) may reflect a favorable treatment response. Given the simplicity and accessibility of serum NSE testing, its incorporation into routine monitoring over longer follow-up periods could provide valuable prognostic information. These findings should be interpreted as hypothesis-generating, rather than definitive biomarker validation.

PRRT demonstrated a favorable safety profile despite the advanced disease status of our cohort. Grade ≥3 toxicities were infrequent and primarily hematologic, including anemia and lymphopenia, consistent with previous reports [23,24]. Severe non-hematologic adverse events were rare, and no cases of myelodysplastic syndrome or therapy-related leukemia were observed during follow-up. No secondary malignancies were observed, further supporting the safety of PRRT in this cohort. In this study, PRRT was generally well tolerated, with a small number of patients requiring treatment discontinuation or reduction of injected activities. One patient developed grade 3 anemia, and another experienced hyperbilirubinemia due to progressive hepatic metastases, underscoring the need for careful monitoring during therapy. Compared with previous reports, including the NETTER-1 trial [25], the incidence of severe hematologic or renal toxicity was low, supporting the safe administration of PRRT in patients with metastatic rectal NET when appropriately monitored. Reduction of injected activity was primarily necessary in patients with renal impairment or extensive bone metastases, highlighting the importance of careful patient selection and individualized dosing. PRRT-related adverse events are generally manageable with appropriate monitoring, including hematological surveillance and dose adjustment when necessary. In addition, renal toxicity can be mitigated by the co-infusion of amino acids containing lysine and arginine, which reduce renal radiation exposure. Careful patient selection, appropriate dose modification, and regular monitoring of hematological parameters are essential to minimize treatment-related toxicities in clinical practice. Patient-specific dosimetry, based on individual imaging and radiopharmaceutical kinetics, may further optimize therapeutic efficacy while minimizing toxicity. However, its routine implementation in clinical practice remains limited and warrants further investigation.

As the largest published dataset of Asian patients with unresectable rectal NETs, our findings offer important real-world insights into clinical outcomes and long-term safety. The NETTER-2 trial has suggested that PRRT may serve as a valuable first-line treatment option for select patients with SSTR-positive NETs [19], and future studies will be essential to clarify its role in earlier treatment settings for unresectable rectal NETs. Patient-specific dosimetry may further optimize therapeutic efficacy and reduce toxicity, although its routine clinical use remains under investigation. In addition, emerging evidence supporting α-particle-based PRRT offers a potential therapeutic strategy for patients who experience disease progression after β-emitter PRRT [26], highlighting a promising future direction for this rare disease subset.

Importantly, our data provide practical insights for gastroenterologists regarding when PRRT may be considered in the treatment algorithm for metastatic rectal NETs.

This study has several limitations. First, the sample size was small, reflecting the rarity of metastatic rectal NETs. Second, the study population was heterogeneous in terms of prior treatments, disease burden, and PRRT regimens, including the use of both ^177^Lu- and ^90^Y-labeled therapies. These factors may have influenced treatment outcomes and limit the generalizability of our findings. Therefore, the results should be interpreted with caution.

## 5. Conclusions

PRRT achieved a median PFS of 18.9 months in Japanese patients with unresectable metastatic rectal NETs, suggesting disease control in this patient population with a favorable safety profile. However, these findings should be interpreted with caution given the small sample size and heterogeneous patient population. To our knowledge, this study represents one of the largest long-term datasets from an Asian population, providing valuable real-world evidence in this understudied disease subset. Although PFS was shorter than that reported for NETs originating from other primary sites, this likely reflects advanced disease status, high tumor burden, and delayed initiation of PRRT in many patients. Baseline NSE elevation and early post-treatment declines may be associated with prognosis and treatment response, suggesting that serum NSE may serve as a simple and clinically accessible biomarker for treatment monitoring. Overall, these findings support PRRT as a clinically meaningful therapeutic option for SSTR-positive unresectable rectal NETs in routine gastroenterology practice and underscore the need for prospective studies evaluating earlier-line use and biomarker-guided strategies.

## Figures and Tables

**Table 1:** Baseline characteristics of patients undergoing PRRT.

Characteristics	*n* = 20
Age at start of PRRT, years, median (range)	63.5 (46–78)
Sex, male (%)	13 (65)
Time from diagnosis to PRRT initiation, months, median (range)	54.7 (6.6–218.6)
ECOG performance status, *n* (%)	
0	17 (85)
1	2 (10)
2	1 (5)
Ki-67 index, %, median (range)	4.5 (1–28.7)
Tumor markers before the start of PRRT, median (range)	
NSE, ng/mL	20.3 (9.7–21.7)
Pro-GRP, pg/mL	46.2 (26.3–79.9)
WHO 2019 grading, *n* (%)	
G1	3 (15)
G2	15 (75)
G3	2 (10)
Non-functional, *n* (%)	20 (100)
Krenning score	
3	3 (15)
4	17 (85)
Primary tumor resection, *n* (%)	13 (65)
R0 resection	12 (60)
R2 resection	1 (5)
Metastatic lesions (%)	
Liver	19 (95)
Bone	11 (55)
Lung	4 (20)
Lymph node	8 (40)
Other	3 (15)
Maximum diameter of liver metastases, mm, median (range) †	38 (21–152)

Abbreviations: PRRT, peptide receptor radionuclide therapy; NSE, neuron-specific enolase; Pro-GRP, Pro-Gastrin-releasing peptide. † Calculated among patients with liver metastases (*n* = 19).

**Table 2:** PRRT treatment characteristics.

	*n* = 20
Institutions, *n* (%)	
Yokohama City University	12 (60)
University Hospital Basel	8 (40)
Radionuclide administered (%)	
[^177^Lu]Lu-DOTA-TATE	12 (60)
[^177^Lu]Lu-DOTA-TOC	5 (25)
Combination of [^90^Y]Y-DOTA-TOC + [^177^Lu]Lu-DOTA-TOC	3 (15)
PRRT cycles, median (range)	3 (1–5)
Number of therapy lines prior to PRRT, median (range)	5 (1–7)
Treatment history prior to PRRT (%)	
SSA	16 (80)
Everolimus	12 (60)
Streptozocin	5 (25)
CAPTEM	2 (10)
Capecitabine	2 (10)
5-FU	5 (25)
Local treatment (%)	
TACE	3 (15)
Radiation therapy (external irradiation)	2 (10)
Heavy ion radiotherapy	1 (5)

Abbreviations: PRRT, peptide receptor radionuclide therapy; SSA, somatostatin analogue; CAPTEM, capecitabine plus temozolomide chemotherapy; 5-FU, 5-Fluorouracil; TACE, transcatheter arterial chemoembolization.

**Table 3:** Treatment efficacy.

	*n* = 20
Median PFS, months (95% CI)	18.9 (13.5–24.3)
Median OS, months (95% CI)	30.3 (18.9–41.7)
ORR, *n* (%)	3 (15)
DCR, *n* (%)	16 (80)
Best overall response (RECIST 1.1), *n* (%)	
CR	0 (0)
PR	3 (15)
SD	13 (65)
PD	3 (15)
Not evaluable (NE)	1 (5)

Abbreviations: ORR, overall response rate; DCR, disease control rate; PFS, progression-free survival; OS, overall survival; CR, complete response; PR, partial response; SD, stable disease; PD, progressive disease.

**Table 4:** Univariate Cox proportional hazards analysis for PFS.

Variable	HR (95% CI)	*p*-Value
*Continuous variables*		
NSE (per 1 ng/mL increase)	1.02 (1.00–1.04)	0.058
Interval from initial diagnosis to PRRT initiation (per 1-month increase)	1.01 (1.00–1.04)	0.29
Age at start of PRRT (per 1-year increase)	1.03 (0.98–1.10)	0.27
Ki-67 index (per 1% increase)	1.02 (0.95–1.10)	0.55
*Categorical variables*		
Sex (male vs. female)	0.81 (0.22–2.91)	0.74
WHO differentiation grade (G1–G2 vs. G3)	1.02 (0.12–8.45)	0.98
Maximum diameter of liver metastases (≤30 mm vs. >30 mm), *n* = 19	0.99 (0.96–1.02)	0.37
Liver tumor burden (≤25% vs. >25%)	0.19 (0.02–1.54)	0.12
Therapy lines prior to PRRT (≤3 vs. >3)	0.64 (0.13–3.21)	0.59
Treatment institution (Basel vs. Yokohama)	0.23 (0.03–1.80)	0.16

Abbreviations: PFS, progression-free survival; HR, hazard ratio; NSE, neuron-specific enolase; PRRT, peptide receptor radionuclide therapy. HR > 1 indicates shorter progression-free survival. WHO grading was based on the 2019 World Health Organization classification.

**Table 5:** Adverse events during PRRT (CTCAE v5.0), *n* = 20.

	All Grades	Grade 1–2	Grade ≥ 3
Hematological toxicities			
Leukopenia	3 (15)	3 (15)	0 (0)
Neutropenia	2 (10)	2 (10)	0 (0)
Lymphopenia	16 (80)	13 (65)	3 (15)
Anemia	16 (80)	15 (75)	1 (5)
Thrombocytopenia	9 (45)	9 (45)	0 (0)
Hepatic/renal laboratory abnormalities			
AST increase	12 (60)	12 (60)	0 (0)
ALT increase	9 (45)	9 (45)	0 (0)
Total Bilirubin increase	1 (5)	0 (0)	1 (5)
Creatinine increase	8 (40)	8 (40)	0 (0)
Non-hematological toxicities			
Nausea	5 (25)	5 (25)	0 (0)
Vomiting	3 (15)	3 (15)	0 (0)
Secondary malignancies			
Myelodysplasia	0 (0)	0 (0)	0 (0)
Leukemia	0 (0)	0 (0)	0 (0)

Data are presented as *n* (%). Abbreviations: PRRT, peptide receptor radionuclide therapy; AST, Aspartate Aminotransferase; ALT, Alanine Aminotransferase.

## Data Availability

The data that support the findings of this study are available from the corresponding author upon reasonable request.
